# 858 The Role of Anatomical Site in Outcomes Following Lower Extremity Burn Injuries

**DOI:** 10.1093/jbcr/iraf019.389

**Published:** 2025-04-01

**Authors:** Christopher Fedor, Hilary Liu, Alexis Henderson, Mare Kaulakis, José Arellano, Garth Elias, Alain Corcos, Jenny Ziembicki, Francesco Egro

**Affiliations:** University of Pittsburgh School of Medicine; University of Pittsburgh Medical Center; University of Pittsburgh School of Medicine; University of Pittsburgh School of Medicine; University of Pittsburgh Medical Center; University of Pittsburgh Medical Center; University of Pittsburgh Medical Center; University of Pittsburgh Medical Center; University of Pittsburgh Medical Center

## Abstract

**Introduction:**

Lower extremity burn wounds present unique challenges due to their susceptibility to complications like graft loss, hypertrophic scarring, and contractures, which can significantly impact functional and aesthetic outcomes. The varying biomechanical properties across different anatomical sites—including shearing forces and pressure points—may influence healing processes. This study aims to identify location-specific risks in lower extremity burn injuries, hypothesizing that joint areas will show higher complication rates compared to non-joint areas, potentially informing targeted management strategies.

**Methods:**

A retrospective review of patients with acute lower extremity burns admitted to an ABA-certified burn center (January 2012-January 2024) was conducted. We collected data on demographics, wound characteristics, surgical interventions, and outcomes. Burns were categorized by anatomical site and grouped into joint-involved (foot, digits, ankle, knee) or non-joint areas (thigh, lower leg). Graft failure, defined as partial or total necrosis of split-thickness skin grafts (STSG), hypertrophic scarring, and contracture rates were compared between these groups.

**Results:**

The study included 507 patients (65.3% male) with a mean age of 39.1 ± 24.2 years. Lower extremity injuries involved: thigh (48.0%), lower leg (48.5%), foot (35.0%), digits (3.5%), ankle (12.1%), knee (16.0%), and entire lower extremity (4.2%). 52.5% of cases underwent surgery. Total graft loss occurred in 3.1% of cases, with no significant difference between joint and non-joint areas (p=0.271). Partial graft loss rates were similar between groups (p=0.993), but the lower leg showed increased risk (p=0.012, OR=2.30, 95% CI [1.20-4.39]). Hypertrophic scarring was more frequent in joint areas (19.6%) compared to non-joint areas (13.7%), though not statistically significant (p=0.077). Contracture rates were significantly higher in joint areas (4.3% vs 0.9%, p=0.017). The ankle had the highest risk of hypertrophic scarring (p=0.001, OR=3.04, 95% CI [1.57–5.90]).

**Conclusions:**

Anatomical location of lower extremity burn injuries significantly affects complication rates, with the lower leg exhibiting a higher incidence of STSG graft loss and joint areas showing increased rates of hypertrophic scarring and contractures. These high-risk locations should be managed pre- and post-operatively with added care to optimize outcomes.

**Applicability of Research to Practice:**

The findings underscore the importance of considering anatomical location in burn management, influencing both surgical decision-making and post-hospital care strategies. Emphasizing location-specific risks to patients may improve compliance with crucial interventions like dressing changes and compression therapy, potentially leading to better outcomes in high-risk areas such as joints and the lower leg.

**Funding for the Study:**

N/A

